# DYNAMIC IMPACTS OF FAMILY CONFLICT ON ELDER ABUSE AND NEGLECT AMONG ADRD CAREGIVERS

**DOI:** 10.1093/geroni/igad104.2017

**Published:** 2023-12-21

**Authors:** Wesley Browning, Jessica Hernandez Chilatra, Mustafa Yildiz, Vicki Winstead, Carolyn Pickering

**Affiliations:** The University of Texas Health Science Center - Houston, Houston, Texas, United States; The University of Alabama at Birmingham, Birmingham, Alabama, United States; University of Alabama at Birmingham, Birmingham, Alabama, United States; University of Alabama at Birmingham, Birmingham, Alabama, United States; University of Alabama at Birmingham, Birmingham, Alabama, United States

## Abstract

Conflict with other family members impacts dementia caregivers’ well-being and stress-appraisals and may also influence their use of elder abuse and neglect (EAN). To test this, we utilized latent class analysis to classify spouse, adult child, grandchild, and nontraditional (step- and in-laws) caregivers (N = 453) based on family conflict levels at baseline. Caregivers were classified into four groups: “low,” “moderate,” “high,” and “EAN accusations.” Spouses were significantly more likely to be in the “low” class compared to other classes. Nontraditional caregivers were more likely to be in the “EAN accusations” class versus the “moderate” class (β = 1.73, SE = .70, p < .05). Belonging to the EAN accusations class predicted EAN at baseline (t = 3.728, p < .001). Next, dynamic structural equation modeling was used to test whether stress from family conflict on a given day predicts use of EAN behaviors. Family conflict stress predicted EAN on the same day (β = 0.05, 95% CI = 0.03-0.07), but not the subsequent day (CL= -0.01, 95% CI = 0.03-0.02). Similar models were conducted for each relationship type. The effect of family conflict stress on EAN was not significant for spouse and grandchildren but remained significant for adult child and non-traditional caregivers. Findings suggest interventions related to conflict management and communication among caregiving families may be beneficial new intervention targets for reducing EAN in ADRD. Addressing family conflict may be most important when the caregiver is an adult child or nontraditional relationship.

